# Determinants of Malaria Vaccine Acceptance: A Systematic Review and Meta‐Analysis of Awareness, Acceptance, Hesitancy, and Willingness to Pay

**DOI:** 10.1002/iid3.70205

**Published:** 2025-05-14

**Authors:** Ganesh Bushi, Mahalaqua Nazli Khatib, Renuka Jyothi. S, Irwanjot Kaur, Abhishek Sharma, Suhaib Iqbal, M. Ravi Kumar, Ashish Singh Chauhan, Teena Vishwakarma, Praveen Malik, Quazi Syed Zahiruddin, Mahendra Pratap Singh, Muhammed Shabil, Rachana Mehta, Sanjit Sah, Hawra Albayat, Tarek Sulaiman, Ali Al bshabshe, Nawal A. Al Kaabi, Hayam A Alrasheed, Mubarak Alfaresi, Amal A. Sabour, Eman Alamri, Maha F. Al‐Subaie, Ali A. Rabaan

**Affiliations:** ^1^ School of Pharmaceutical Sciences Lovely Professional University Phagwara Punjab India; ^2^ Division of Evidence Synthessis, Global Consortium of Public Health and Research Datta Meghe Institute of Higher Education Wardha Maharashtra India; ^3^ Department of Biotechnology and Genetics School of Sciences, JAIN (Deemed to be University) Bangalore Karnataka India; ^4^ Department of Allied Healthcare and Sciences Vivekananda Global University Jaipur Rajasthan India; ^5^ Department of Medicine NIMS University Jaipur Rajasthan India; ^6^ Chandigarh Pharmacy College, Chandigarh Group of College, Jhanjeri Mohali Punjab India; ^7^ Department of Chemistry Raghu Engineering College Visakhapatnam Andhra Pradesh India; ^8^ Division of Research and Innovation Uttaranchal Institute of Pharmaceutical Sciences, Uttaranchal University Dehradun Uttarakhand India; ^9^ IES Institute of Pharmacy IES University Bhopal Madhya Pradesh India; ^10^ New Delhi Institute of Management Delhi India; ^11^ South Asia Infant Feeding Research Network, Division of Evidence Synthesis, Global Consortium of Public Health and Research Datta Meghe Institute of Higher Education Wardha Maharashtra India; ^12^ Center for Global Health Research, Saveetha Medical College and Hospital, Saveetha Institute of Medical and Technical Sciences Saveetha University Chennai Tamil Nadu India; ^13^ University Center for Research and Development, Chandigarh University Mohali Punjab India; ^14^ Medical Laboratories Techniques Department AL‐Mustaqbal University Hillah Babil Iraq; ^15^ Clinical Microbiology, RDC Manav Rachna International Institute of Research and Studies Faridabad Haryana India; ^16^ Dr Lal PathLabs ‐ Nepal Maharajgunj Kathmandu Nepal; ^17^ Department of Pediatrics, Dr. D. Y. Patil Medical College, Hospital and Research Centre Dr. D. Y. Patil Vidyapeeth (Deemed‐to‐be‐University), Pimpri Pune India; ^18^ Department of Public Health Dentistry, Dr. D.Y. Patil Dental College and Hospital Dr. D. Y. Patil Vidyapeeth (Deemed‐to‐be‐University), Pimpri Pune Maharashtra India; ^19^ SR Sanjeevani Hospital Kalyanpur Siraha Nepal; ^20^ Infectious Disease Department King Saud Medical City Riyadh Saudi Arabia; ^21^ Infectious Diseases Section, Medical Specialties Department King Fahad Medical City Riyadh Saudi Arabia; ^22^ Adult critical care Department of medicine, Division of adult critical care, College of medicine King Khalid University Abha Saudi Arabia; ^23^ College of Medicine and Health Science Khalifa University Abu Dhabi United Arab Emirates; ^24^ Sheikh Khalifa Medical City Abu Dhabi Health Services Company (SEHA) Abu Dhabi United Arab Emirates; ^25^ Department of Pharmacy Practice, College of Pharmacy Princess Nourah bint Abdulrahman University Riyadh Saudi Arabia; ^26^ Department of Microbiology National Reference Laboratory, Cleveland Clinic Abu Dhabi Abu Dhabi United Arab Emirates; ^27^ Department of Pathology, College of Medicine Mohammed Bin Rashid University of Medicine and Health Sciences Dubai United Arab Emirates; ^28^ Department of Botany and Microbiology, College of Science King Saud University Riyadh Saudi Arabia; ^29^ Food Science and Nutrition, Faculty of Science University of Tabuk Tabuk Saudi Arabia; ^30^ Research Center Dr. Sulaiman Alhabib Medical Group Riyadh Saudi Arabia; ^31^ College of Medicine Alfaisal University Riyadh Saudi Arabia; ^32^ Molecular Diagnostic Laboratory, Johns Hopkins Aramco Healthcare Dhahran Saudi Arabia; ^33^ Department of Public Health and Nutrition The University of Haripur Haripur Pakistan

**Keywords:** acceptance, awareness, hesitancy, malaria vaccine, systematic review, willingness to pay

## Abstract

**Background:**

Malaria is a life‐threatening disease caused by Plasmodium parasites, transmitted through the bites of infected female Anopheles mosquitoes. It remains a major global health issue, with 263 million cases and 597,000 deaths in 2023, primarily affecting young children and pregnant women. This review evaluates awareness, acceptance, hesitancy, and willingness to pay (WTP) for the RTS,S/AS01 malaria vaccine, along with the key factors influencing these outcomes.

**Methods:**

A comprehensive literature search was conducted in Web of Science, PubMed, and Embase, covering publications up to 18 June 2024. Observational studies assessing awareness, acceptance, hesitancy, and WTP for the malaria vaccine in endemic regions were included. Two independent reviewers screened the studies. Data extraction was performed using Nested Knowledge software and analyzed with R v.4.4. Pooled prevalences were estimated using random‐effects models, and heterogeneity was assessed with the I² statistic.

**Results:**

Eighteen studies with 21,975 participants provided insights into malaria vaccine dynamics: 32% awareness (95% CI, 18%–50%), 83% acceptance (95% CI, 75%–89%), 14% hesitancy (95% CI, 7%–26%), and 58% WTP (95% CI, 34%–79%). Key determinants of acceptance included age, where younger adults (18–24 years) showed lower acceptance (OR = 0.64, 95% CI, 0.35–0.93). Employment, particularly farmers, had higher acceptance rates (OR = 3.20, 95% CI, 1.00–7.40). Lower socioeconomic status and larger family sizes were associated with decreased acceptance (OR = 0.18, 95% CI, 0.02–0.38).

**Conclusion:**

This review revealed an 83% acceptance rate for the malaria vaccine, with variability in awareness (32%), hesitancy (14%), and willingness to pay (58%). Age, employment, and socioeconomic status were significant determinants of acceptance. However, due to potential publication bias and high heterogeneity, these findings should be cautiously interpreted. The results highlight the necessity for targeted interventions to enhance vaccine acceptance. Further research is required to elucidate factors that influence vaccine acceptance.

## Graphical abstract

This review revealed an 83% acceptance rate for the malaria vaccine, with variability in awareness (32%), hesitancy (14%), and willingness to pay (58%). Age, employment, and socioeconomic status were significant determinants of acceptance. Further research is needed to examine additional factors influencing vaccine acceptance.

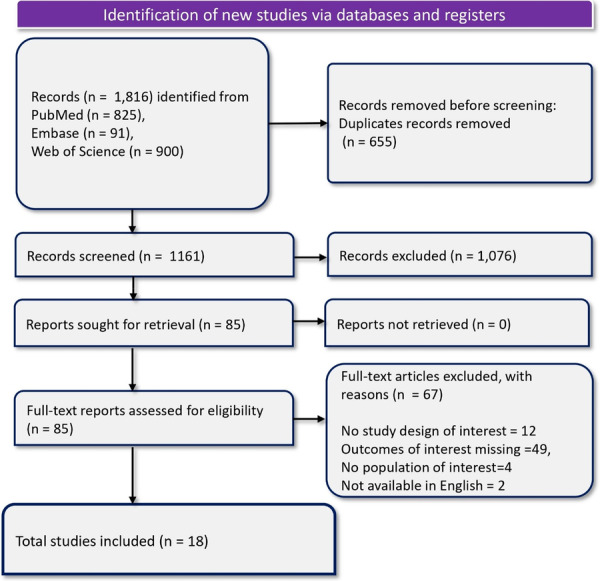

## 1 Background

Malaria is a life‐threatening infectious disease caused by *Plasmodium* parasites, transmitted through the bites of infected female Anopheles mosquitoes. It is characterized by symptoms such as fever, chills, and flu‐like illness and can lead to severe complications or death if untreated [1]. Malaria predominantly affects tropical and subtropical regions, with Sub‐Saharan Africa bearing the highest burden [2]. The disease transmission cycle begins when an infected mosquito bites a human, introducing the parasite into the bloodstream, where it travels to the liver, matures, and then infects red blood cells [3]. Despite advances in vector control, diagnostics, and treatment, malaria remains a major global health challenge [4, 5].

The World Malaria Report 2024 by the World Health Organization (WHO) reported an estimated 263 million cases and 597,000 malaria deaths worldwide in 2023, with children under five being the most vulnerable [6]. Approximately 95% of the deaths occurred in the WHO African Region, where many at risk still lack access to the services needed to prevent, detect, and treat the disease. The ongoing burden highlights the need for additional interventions, such as the RTS,S/AS01 (RTS, S) malaria vaccine, to complement existing control strategies.

The RTS,S, malaria vaccine, branded as Mosquirix®, emerged as a promising candidate for malaria prevention, receiving approval from the WHO in October 2021 for use in children in regions with moderate to high malaria transmission. This marked a significant milestone in the global effort to combat malaria [7]. Clinical trials have demonstrated its efficacy in reducing clinical and severe malaria cases among young children. However, the success of malaria vaccination programs depends not only on the vaccine's efficacy but also on its acceptance, awareness, and WTP among target populations. Vaccine acceptance and hesitancy are critical factors influencing acceptance [8, 9]. Alongside RTS,S, the WHO has also introduced the R21/Matrix‐M malaria vaccine, which shows promising efficacy. However, given the widespread deployment of RTS,S, which has been integrated into national immunization programs in several African countries, this review primarily focuses on RTS,S to assess its acceptance and associated factors [10]. Vaccine acceptance is driven by determinants such as perceived disease risk, vaccine benefits, trust in healthcare providers, and sociocultural beliefs [11, 12, 13]. In contrast, vaccine hesitancy—defined by the WHO as a “delay in acceptance or refusal of vaccines despite the availability of vaccination services”—remains a significant barrier to achieving high vaccination coverage [14]. Hesitancy is influenced by factors such as misinformation, fear of side effects, religious or philosophical beliefs, and distrust in healthcare systems. Understanding these determinants is essential for developing effective communication and intervention strategies to enhance vaccine acceptance [15].

The integration of a malaria vaccine into national immunization programs requires understanding key determinants such as vaccine acceptance, hesitancy, awareness, and WTP. This systematic review and meta‐analysis provide evidence to guide policymakers, health practitioners, and researchers in creating effective vaccination strategies. Awareness of the malaria vaccine and its benefits is a precursor to acceptance and WTP [16, 17]. Public health campaigns and community engagement play crucial roles in disseminating information and educating populations about vaccination importance. In many malaria‐endemic regions, targeted awareness programs that address local concerns and misconceptions are essential to foster a supportive environment for vaccination efforts [18]. Additionally, WTP for vaccines is crucial, particularly in low‐ and middle‐income countries where out‐of‐pocket health expenditures are common. For instance, while routine childhood vaccines such as DTP and polio are often provided free of charge, there are instances where additional vaccines, such as the human papillomavirus vaccine, require out‐of‐pocket payment, especially in low‐income countries where healthcare funding may be limited. These payments can vary depending on the local health system and economic conditions. WTP studies provide insights into the perceived value of the vaccine and financial barriers to access, helping inform pricing strategies and subsidy policies to ensure vaccines are affordable and accessible to all socioeconomic groups [19]. A previous systematic review by Suleiman et al. (2022) examined malaria vaccine acceptance in endemic regions, reporting higher acceptance rates both among the general population and mothers [20]. Despite these valuable insights, significant gaps remain in understanding the factors influencing vaccine acceptance, including awareness, hesitancy, and willingness to pay. Addressing these gaps is crucial for developing strategies to improve vaccine acceptance in malaria‐endemic areas.

This review sought to address several critical questions: What is the level of awareness regarding the malaria vaccine across different populations? What is the prevalence of vaccine acceptance and hesitancy? What factors influence vaccine acceptance? Although the initial aim was to explore determinants of awareness, acceptance, hesitancy, and WTP, the data from the included studies predominantly addressed factors related to vaccine acceptance. Therefore, this meta‐analysis focuses on acceptance‐related determinants, while recognizing the need for further research on awareness, hesitancy, and WTP. By addressing these questions, the review aims to enhance understanding of vaccine acceptance dynamics and support global malaria control and eradication efforts. The objective is to provide evidence‐based insights to inform targeted interventions and policy decisions aimed at improving malaria vaccine coverage in endemic regions.

## 2 Methods

This review was conducted following the Preferred Reporting Items for Systematic Reviews and Meta‐Analyses 2020 (PRISMA‐2020) guidelines [21] (Table S1). The review protocol has been registered with PROSPERO (CRD42024555552). Nested knowledge software was used to manage the complete data for the review.

### 2.1 Eligibility Criteria

Studies were included based on the following criteria: The population consisted of individuals of any age from malaria‐endemic regions, with exposure to information or programs related to the malaria vaccine. The outcomes of interest included awareness, acceptance, hesitancy, and WTP for the malaria vaccine, as well as key determinants influencing these outcomes. Observational studies, including cross‐sectional, cohort, and case‐control designs, were considered. Only studies published in English were included, while those that did not provide specific data on the malaria vaccine or were reviews, editorials, commentaries, or case reports, were excluded (Table S2).

### 2.2 Literature Search

To identify relevant studies, an extensive literature search was conducted across several electronic databases, including Web of Science, PubMed, and Embase, from the inception of these databases up to June 18, 2024. The search strategy combined MeSH terms and keywords related to the malaria vaccine, such as awareness, acceptance, hesitancy, willingness to pay, and determinants. Specific search terms included “malaria vaccine,” “RTS,S vaccine,” “awareness,” “acceptance,” “hesitancy,” and “willingness to pay.” Additionally, manual searches of reference lists from included studies and other relevant literature were performed to ensure no pertinent studies were overlooked (Table S3).

### 2.3 Screening and Data Extraction

The screening process was conducted in two phases: an initial review of titles and abstracts, followed by a full‐text review using Nested Knowledge software. In the first phase, two independent reviewers evaluated the titles and abstracts of all retrieved records to determine potential eligibility. Any disagreements between the reviewers were resolved through discussions with a third reviewer. In the second phase, the full texts of the potentially eligible studies were obtained and assessed by the same two independent reviewers. Studies that met the eligibility criteria were included in the review, with any discrepancies resolved in the same manner as during the first phase.

Relevant information was collected from each included study. Tags were created in Nested Knowledge to extract data such as study characteristics (author, year of publication, country, and study design), population characteristics (including sample size, age, and gender), details on awareness, acceptance, hesitancy, and WTP for the malaria vaccine, and key determinants vaccine acceptance.

### 2.4 Quality Assessment

The methodological quality of the studies was evaluated using a modified version of the Newcastle‐Ottawa Scale (NOS), with a scoring range from 0 to 6, tailored for malaria‐related research. The assessment criteria included the representativeness of the study sample (0–2 points), the adequacy of sample size (0–1 point), the precision of the malaria definition (0‐1 point), and the accuracy in identifying associated health outcomes (0–1 point). Studies scoring 4 to 6 were categorized as low risk of bias, indicating high methodological quality. Those scoring 2 to 3 were considered moderate risk, and scores of 0 to 1 were classified as high risk, suggesting lower methodological quality [22].

### 2.5 Statistical Analysis

Meta‐analysis was conducted using R® software (version 4.4) with the “meta” and “metafor” packages. A random‐effects model was employed to account for potential heterogeneity in pooling prevalence data. The inverse variance method was used to pool logit‐transformed proportions (PLOGIT), which were calculated by dividing the number of events (e.g., vaccine acceptance, awareness) by the sample size of each study. The results were presented as proportions, with the corresponding 95% confidence intervals (CIs) shown in the forest plot. For clarity, the proportions were converted to whole numbers in the text for easy interpretation [23]. For the odds ratio (OR), we used the metagen R package to pool ORs from the included studies. These ORs were directly extracted from the individual studies, and the pooled ORs with their corresponding 95% CIs were calculated. The OR results were visually represented through forest plots, illustrating effect sizes and their respective confidence intervals. Heterogeneity among the studies was assessed using the I² statistic [24, 25].

Leave‐one‐out sensitivity analyses were conducted to examine the robustness of the results by sequentially excluding each study and re‐conducting the analysis to identify potential influences on the overall findings. A Doi plot and the Luis Furuya‐Kanamori (LFK) index were utilized to assess publication bias; while there is no strict minimum number of studies required to construct a Doi plot, it is generally recommended to include at least 10 to ensure an effective evaluation of publication bias. The Doi plot visually examines the symmetry of effect sizes against their precision, where any observed asymmetry may indicate publication bias. The LFK index quantifies this asymmetry: values close to zero indicate negligible asymmetry, values between 1 and 2 suggest moderate asymmetry, and values of 2 or greater denote significant asymmetry [26, 27].

## 3 Results

### 3.1 Literature Search

**FIGURE 1:**
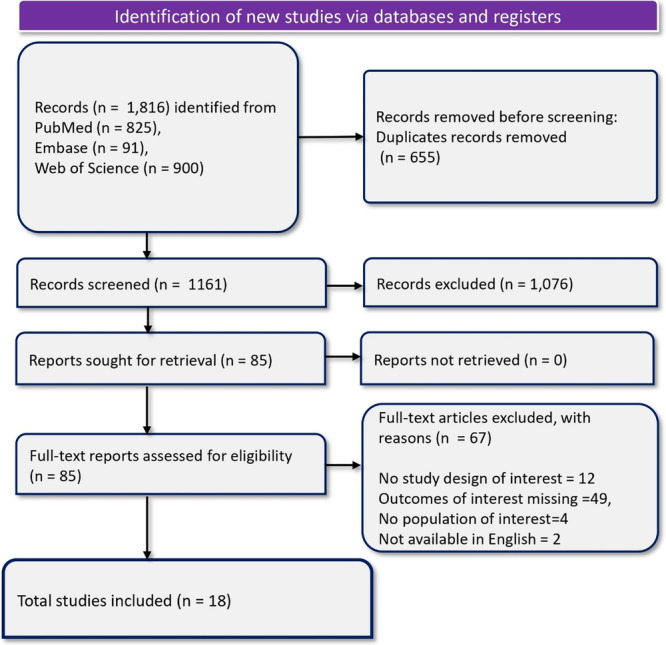
PRISMA flow diagram.

A total of 1816 records were identified from PubMed (825), Embase (91), and Web of Science (900). After removing 655 duplicates, 1161 records were screened, resulting in the exclusion of 1076 records. Eighty‐five records were retrieved for full‐text screening, and 67 were excluded for the following reasons: no study design of interest (*n* = 12), outcomes of interest missing (*n* = 49), no population of interest (*n* = 4), and two studies were not available in English. Eighteen studies were eligible for final inclusion in the review. Figure 1 PRISMA flow diagram, depicts the literature search results of the review.

### 3.2 Summary of Study Characteristics

The included studies encompass a total of 21,975 individuals, with 5894 males, and sample sizes ranging from 347 to 5502 participants. The common study populations focused on caregivers, parents, and adult residents responsible for young children, particularly caregivers of children under 5 years old (Table 1). A total of 16 studies reported on vaccine acceptance, 10 on hesitancy, 14 on awareness, 6 on WTP, and 9 studies reported various factors associated with malaria vaccine acceptance. The age of participants varied, with means ranging from 19.53 years to 39 years. Most studies employed a cross‐sectional design, with one prospective design conducted across several countries, including six from Nigeria [28, 29, 33, 34, 35, 39], four from Ghana [36, 37, 38, 45], two from Ethiopia [31, 44], and one each from Uganda [32], Kenya [41], Bangladesh [30], the Democratic Republic of Congo [40], Guinea, Sierra Leone [42], and Tanzania [43]. Participants were categorized into two groups: caregivers of children, who were responsible for the care of children under five, and self‐respondents, who reported on their own vaccination awareness, acceptance, or willingness to pay without caregiving responsibilities. The modified NOS revealed a moderate to high quality of the included studies (Table S4).

**TABLE 1:** Summary characteristics of included studies.

Study	Country	Study design	Study population	No. of males	Age (Mean ± SD) (years)	Sample size
Abdulkadir 2015 [28]	Nigeria	Cross‐sectional study	Caregivers of children under 5 years old and opinion leaders in five communities in Ibadan North LGA	11	29.8 (5.8)	427
Ajayi 2023 [29]	Nigeria	Cross‐sectional study	Caregivers of under 5 children	90	33.5 (8.5)	504
Amin 2023 [30]	Bangladesh	Cross‐sectional study	Parents of children under 5; participants from malaria endemic areas	189	>18 years	405
Asmare 2022 [31]	Ethiopia	Cross‐sectional study	Caregivers of under‐5 children	77	32.7 (6.6)	406
Bongomin 2024 [32]	Uganda	Cross‐sectional study	Adults with children 5 years or younger residing in Pece–Laroo division, Gulu City	119	32.4 (10.6)	432
Chinawa 2024 [33]	Nigeria	Cross‐sectional study	Mothers recruited from outpatient and immunization clinics in Enugu metropolis	240	32.0 (8)	491
Chukwuocha 2018 [34]	Nigeria	Prospective study	Caregivers (mostly mothers)	NA	19.53 (7.24)	500
Emmanuel 2024 [35]	Nigeria	Cross‐sectional study	Caregivers of under 5‐year‐old children	180	NA	347
Febir 2013 [36]	Ghana	Cross‐sectional study	Children enrolled in Kintampo North and South districts for RTS,S malaria vaccine trials	NA	19–55 years	466
Hussein 2024 [37]	Ghana	Cross‐sectional study	Parents of children eligible for vaccination	247	36.0 median, (IQR:31.0–41.0)	765
Immurana 2022 [38]	Ghana	Cross‐sectional study	Women aged 15–49 residing in selected households	1547	15–49 years	3004
Kabir Sulaiman 2023 [39]	Nigeria	Cross‐sectional study	Adult residents (> = 18 years) who care for children under five	1961	30 (9.2)	3389
Nyalundja 2024 [40]	Democratic Republic of Congo	Cross‐sectional study	Adult population of Bukavu	787	39 (median, IQR:26–54)	1612
Ojakaa 2014 [41]	Kenya	Cross‐sectional study	Caregivers of sick children	129	NA	1997
Röbl 2023 [42]	Guinea and Sierra Leone	Cross‐sectional study	Caregivers for children under 5 years of age	NA	NA	702
Romore 2015 [43]	Tanzania	Cross‐sectional study	Parents of children, especially mothers	NA	NA	5502
Wagnew 2021 [44]	Ethiopia	Cross‐sectional study	Caregivers of under‐five children	86	18–46 years	604
Yeboah 2022 [45]	Ghana	Cross‐sectional study	Children 6–24 months in Kassena Nankana Municipality	231	27 (5)	422

## 4 Findings of Meta‐Analysis

### 4.1 Awareness of Malaria Vaccine

**FIGURE 2:**
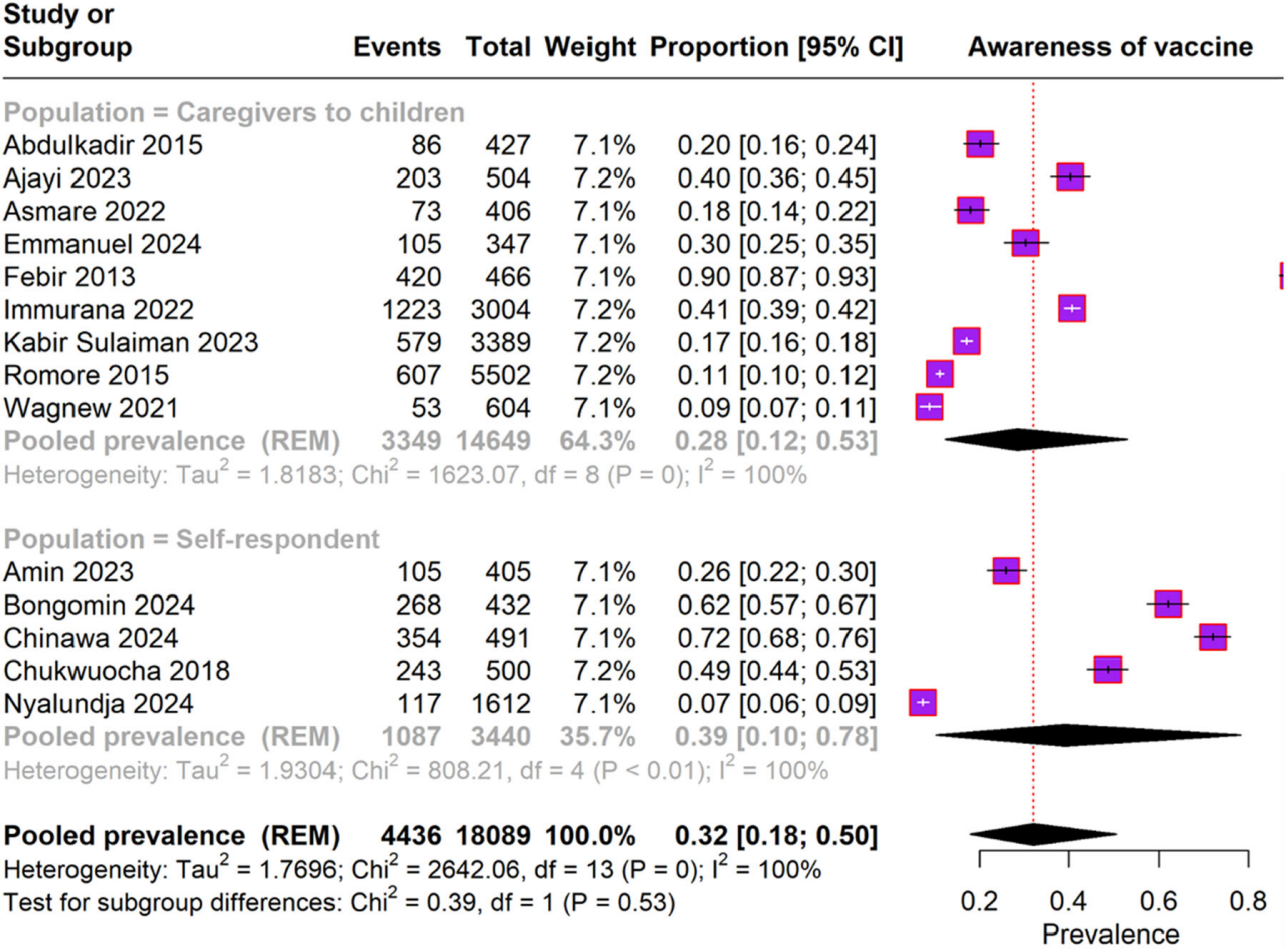
Forest plot representing the awareness rate of malaria vaccine.

The meta‐analysis analysis included 14 studies with a total of 3440 participants. The overall pooled prevalence of malaria vaccine awareness was 32% (95% CI, 18%–50%), with significant heterogeneity (I² = 100%) (Figure 2). For caregivers of children, the pooled prevalence of vaccine awareness was 28% (95% CI, 12%–53%), also showing substantial heterogeneity (I² = 100%). In the subgroup of self‐respondents, the pooled prevalence of vaccine awareness was 39% (95% CI, 10%–78%), again with significant heterogeneity (I² = 100%).

### 4.2 Acceptance of Malaria Vaccine

**FIGURE 3:**
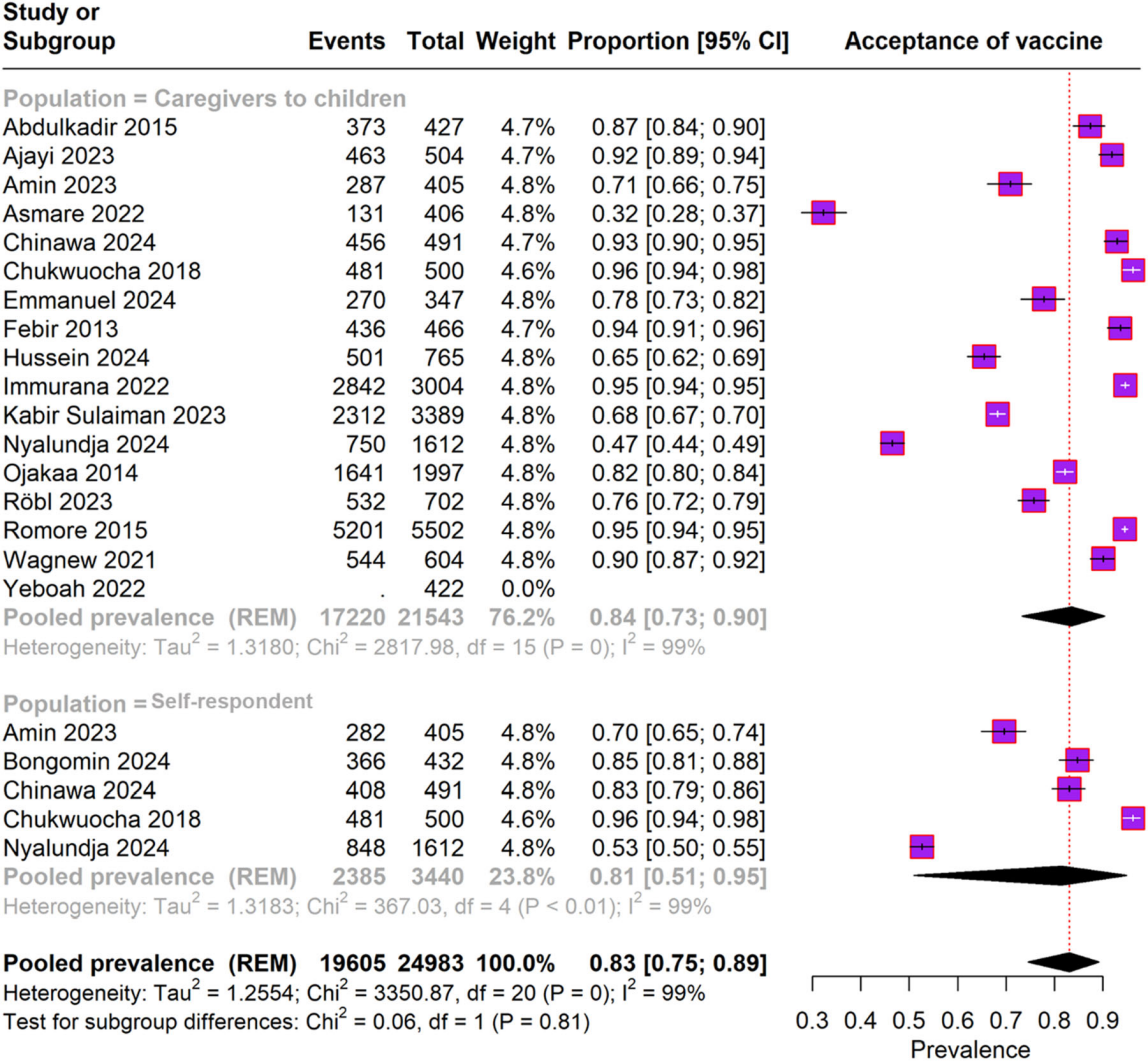
Forest plot depicting the acceptance rate of malaria vaccine.

Eighteen studies involving 3440 participants reported the malaria vaccine acceptance rate (Figure 3). The overall pooled prevalence of vaccine acceptance was 83% (95% CI, 75%–89%), with significant heterogeneity (I² = 99%). Among caregivers of children, the pooled prevalence of vaccine acceptance was 84% (95% CI, 73%–90%), also showing substantial heterogeneity (I² = 99%). In the self‐respondent subgroup, the pooled prevalence was 81% (95% CI, 51%–95%), with similar heterogeneity (I² = 99%).

### 4.3 Hesitancy Toward the Malaria Vaccine

**FIGURE 4:**
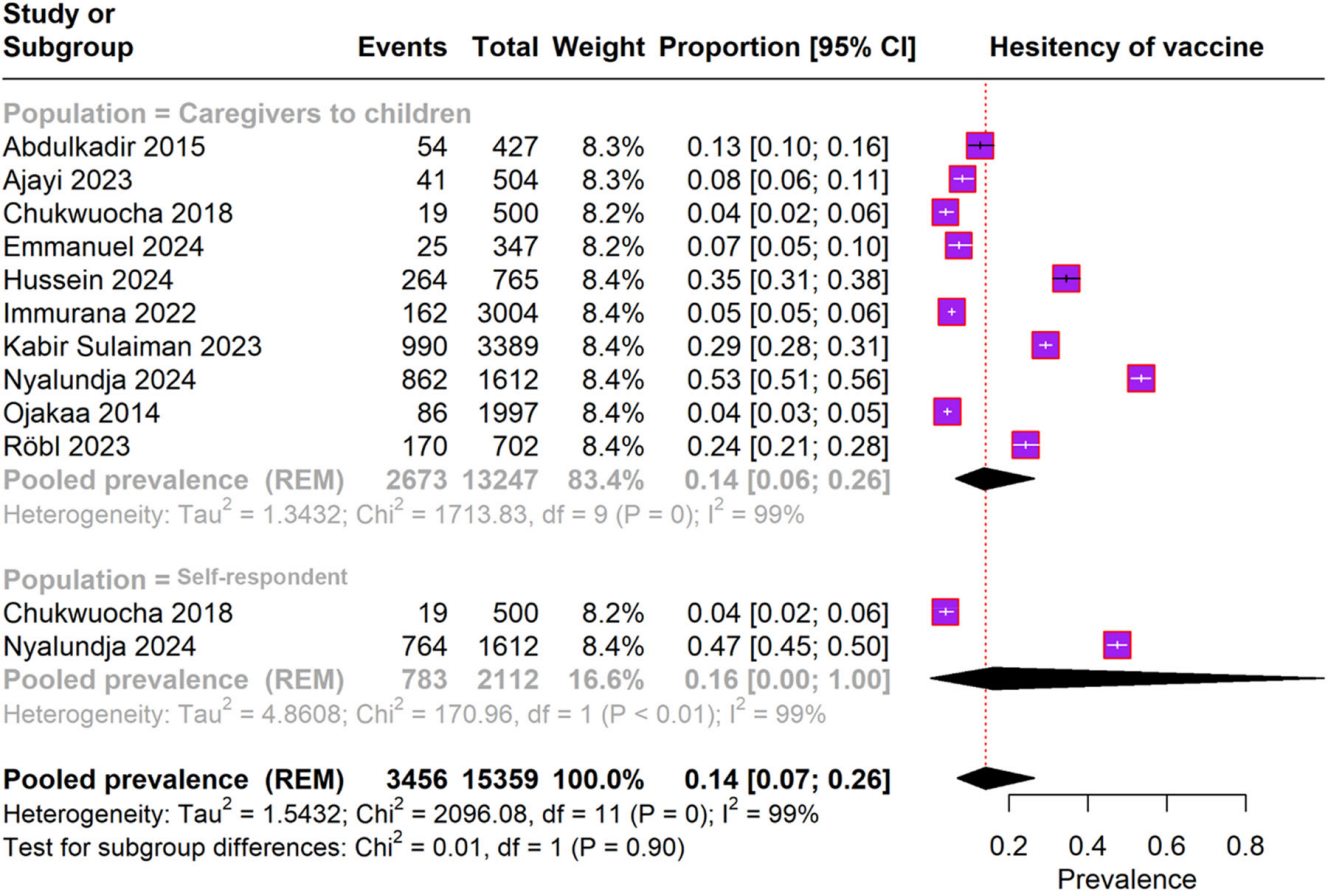
Forest plot depicting the hesitancy rate of malaria vaccine.

The forest plot in Figure 4 includes data from 10 studies involving 15,359 participants reporting on malaria vaccine hesitancy. The overall pooled prevalence of vaccine hesitancy was 14% (95% CI, 7%–26%), with significant heterogeneity (I² = 99%). For caregivers of children, the pooled prevalence of vaccine hesitancy was also 14% (95% CI, 6%–26%), exhibiting substantial heterogeneity (I² = 99%). In the self‐respondent subgroup, the pooled prevalence of vaccine hesitancy was 16% (95% CI, 0.9%–100%), with similar heterogeneity (I² = 99%).

### 4.4 Willingness to Pay for Malaria Vaccine

**Figure 5:**
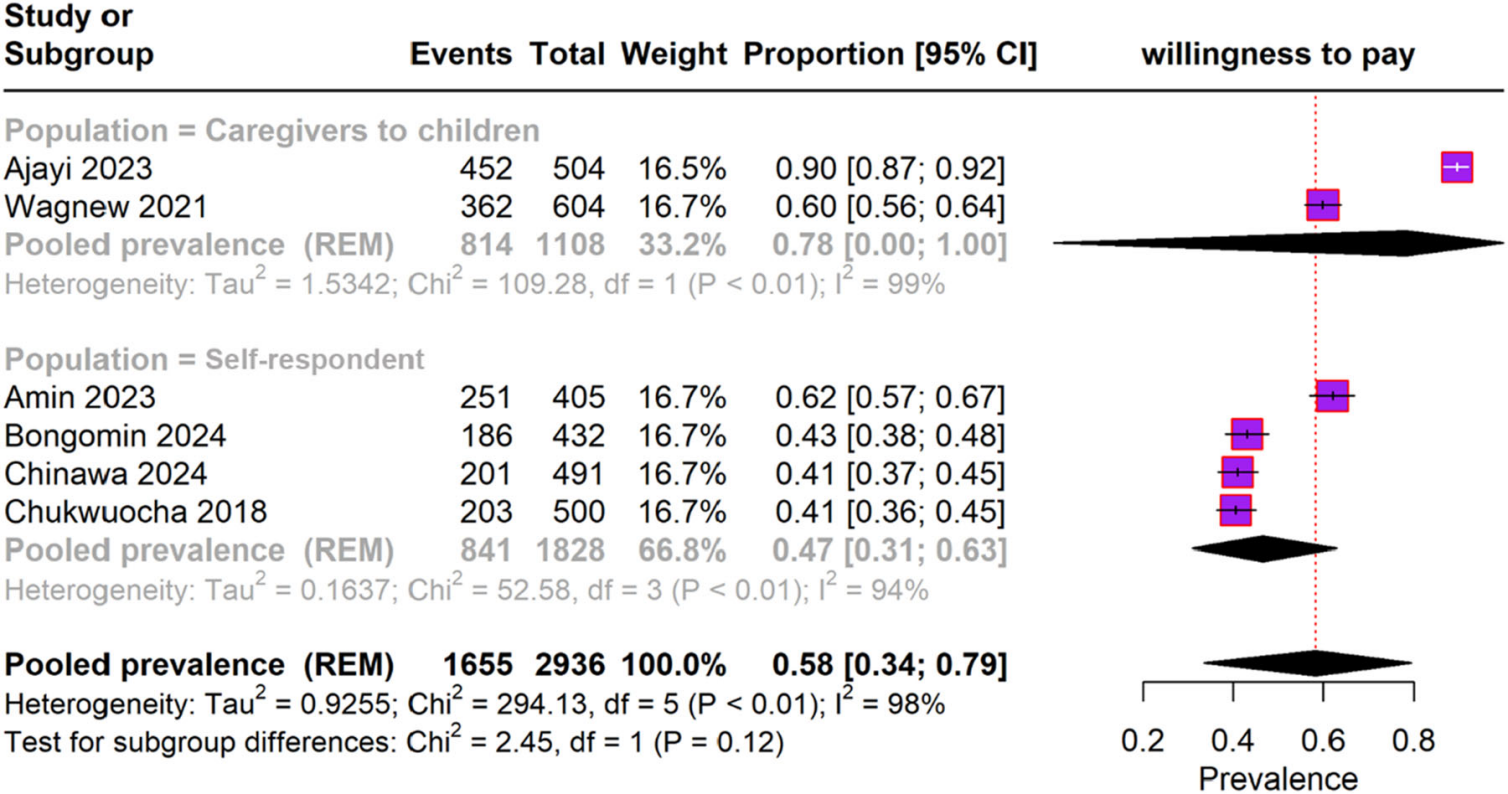
Forest plot depicting the WTP for malaria vaccine.

The pooled prevalence of WTP for the malaria vaccine across different populations, as depicted in forest plot Figure 5, included six studies with a total of 2936 participants. The overall pooled prevalence of WTP for the malaria vaccine was 58% (95% CI, 34%–79%), with significant heterogeneity (I² = 98%). Among caregivers of children, two studies reported a pooled prevalence of WTP of 78% (95% CI, 80%–100%), with substantial heterogeneity (I² = 99%). In the self‐respondent subgroup, four studies reported a pooled prevalence of WTP of 47% (95% CI, 31%–63%), with significant heterogeneity (I² = 94%).

### 4.5 Factors Influencing Malaria Vaccine Acceptance

The meta‐analysis identified significant variability in malaria vaccine acceptance across different demographics and socioeconomic statuses (Table 2). Higher educational levels increased acceptance, with tertiary education showing the highest odds (OR, 4.09; 95% CI, −0.02 to 8.20). Employment status also influenced acceptance, with employed individuals more likely to accept the vaccine (OR, 2.28; 95% CI, 0.69–3.87). Specific occupations, such as farming (OR, 3.20; 95% CI, 1.00–7.40), showed higher acceptance rates. Health history played a role, as caregivers who had malaria in the past year showed higher acceptance (OR, 1.57; 95% CI, 0.74–2.4). Higher‐income levels were linked to greater acceptance (OR, 2.24; 95% CI, 1.17–3.30). Married individuals (OR, 1.18; 95% CI, 0.84–3.04) were likely to accept the vaccine. Perceived susceptibility to malaria (OR, 26.90; 95% CI, 6.15–47.65) significantly increased acceptance. In contrast, larger family sizes ( >10 members) were associated with lower acceptance (OR, 0.18; 95% CI, 0.02–0.38). Younger individuals (18–24 years) were less likely to accept the vaccine compared to those over 24 years (OR, 0.64; 95% CI, 0.35–0.93). Lower socioeconomic status and household wealth were associated with lower acceptance, with the lowest wealth index showing an OR of 0.10 (95% CI, 0.00–0.20) (Figure S1).

**TABLE 2:** Factors influencing vaccine acceptance.

Category	Factors	No. of studies	Heterogeneity (I^2^)	OR (95% CI)
Age	18–24 yr versus > 24 yr	1	NA	0.64 (0.35; 0.93)
	26–35 yr versus < 26 yr	1	NA	1.40 (0.49; 2.30)
	36–65 yr versus < 26 yr	1	NA	1.48 (0.33; 2.63)
	Over 64 years versus < 64 yr	1	NA	2.07 (0.65; 3.48)
Education	Primary education versus No education	1	NA	2.55 (−1.71; 6.81)
	Secondary education versus No education	1	NA	1.85 (0.29; 3.41)
	Tertiary education versus No education	1	NA	4.09 (−0.02; 8.20)
	Level of education ‐ Low versus High	1	NA	0.61 (0.13; 1.08)
	Level of education ‐ Medium versus High	1	NA	0.84 (0.58; 1.09)
Employment	Employed versus Unemployed	1	NA	2.28 (0.69; 3.87)
	Occupation Business versus Other occupations	1	NA	1.01 (−0.39; 2.41)
	Occupation Farmer versus Other occupations	1	NA	3.20 (1.00; 7.40)
	Occupation Day laborers versus Other occupations	1	NA	0.62 (−0.23; 1.47)
	Occupation Housewife versus Other occupations	1	NA	1.20 (−0.24; 2.64)
Family size	Family Size > 10 versus < = 10	1	NA	0.18 (0.02; 0.38)
	Family Size 5–10 versus < 5	1	NA	0.82 (0.13; 1.51)
Gender	Male versus Female	2	40	0.78 (0.08; 1.48)
Health and vaccine experience	Caregiver had malaria in last 1 year versus No malaria	2	0	1.57 (0.74; 2.4)
	Child had malaria in last 1 years versus No malaria	1	NA	3.07 (0.43; 5.70)
Income	Avg monthly income in NGN > 100,000 versus < 30,000	1	NA	2.24 (1.18; 3.31)
Knowledge and perception	Educated on malaria vaccine versus Not educated	2	80	1.51 (−1.4; 4.43)
	Perceived susceptibility to malaria versus No susceptibility	1	NA	26.90 (13.2; 54.7)
Location	Urban location versus Rural	3	0	0.97 (0.641; 1.300)
Marital status	Married versus Unmarried	4	0	1.183 (0.84; 1.526)
Nationality	Nationality ‐ Native versus Nonnative	1	NA	1.27 (0.01; 2.52)
Socioeconomic status	Socioeconomic status Low versus High	1	NA	0.20 (0.00; 0.40)
	Household wealth index ‐ 5th quintile versus 1st quintile	1	NA	0.10 (0.00; 0.20)
	Household wealth index ss ‐ 4th quintile versus 1st quintile	1	NA	0.34 (0.05; 0.63)
	Household wealth index ‐ 2nd quintile versus 1st quintile	1	NA	0.49 (0.08; 0.89)

### 4.6 Sensitivity Analysis

The forest plot in Figure S2 depicts the leave‐one‐out meta‐analysis, which evaluates the robustness of the pooled prevalence estimate of malaria vaccine acceptance by sequentially omitting each study. The pooled prevalence of malaria vaccine acceptance for all studies was 83% (95% CI, 74%–89%). When each study was omitted one at a time, the pooled prevalence estimates ranged from 81% (95% CI, 73%–88%) when Chukwuocha [34] was omitted, to 84% (95% CI, 76%–89%) when Nyalundja (2024) [40] was omitted.

### 4.7 Publication Bias

The funnel plot presented assesses the publication bias in the studies included in the meta‐analysis; the LFK index is 5.3 (Figure S3). The plot reveals a significant asymmetry, with a concentration of studies showing smaller effect sizes and higher Z‐scores, indicating a potential overestimation of the prevalence of vaccine acceptance due to publication bias. This finding underscores the importance of interpreting the results with caution, as the presence of publication bias may influence the overall conclusions drawn from the meta‐analysis.

## 5 Discussion

### 5.1 General Interpretation of the Results in the Context of Other Evidence

The findings from this systematic review and meta‐analysis offer a comprehensive perspective on the determinants influencing malaria vaccine awareness, acceptance, hesitancy, and WTP. By synthesizing data across diverse settings and populations, this study provides valuable insights into the multifaceted nature of vaccine acceptance and the challenges that need to be addressed to enhance vaccination efforts globally. The meta‐analysis revealed a low level of awareness regarding the malaria vaccine, with a pooled prevalence of 32%. This figure was notably higher among populations with better access to health information and education, highlighting the critical role that public health campaigns and community engagement play in improving vaccine acceptance. The highest awareness was observed in a study by Chinawa et al. [33], which reported an awareness level of 72.1%. This finding underscores the effectiveness of well‐targeted educational initiatives in overcoming misconceptions and fostering greater acceptance of the malaria vaccine.

Vaccine hesitancy emerged as a significant barrier, with a pooled prevalence of 14%. Hesitancy peaked at 53.5% in the study by Nyalundja [40], driven largely by misinformation, fear of side effects, and distrust in healthcare systems. These factors highlight the urgent need for strategies that build trust and effectively communicate the benefits and safety of the malaria vaccine. The role of healthcare providers in dispelling myths and addressing concerns is particularly important, as their endorsement can significantly influence public perception and acceptance of the vaccine.

Despite these challenges, the overall acceptance of the malaria vaccine was high, with a pooled prevalence of 83.1%. Acceptance was influenced by several factors, including age, education, and employment status. Notably, younger individuals (aged 18–24 years) showed lower acceptance rates, which may be linked to perceived invulnerability or a lack of awareness about the risks of malaria. While this age group is not eligible for vaccination, their attitudes may influence vaccine acceptance within the broader community, particularly among caregivers of young children. Education about the vaccine emerged as a key determinant of acceptance, with individuals who were informed about the malaria vaccine being more likely to accept it, although this was not statistically significant (OR, 1.51). Perceived susceptibility to malaria also played a crucial role, with those recognizing their vulnerability to the disease exhibiting substantially higher odds of vaccine acceptance (OR, 26.90). Additionally, married individuals were more inclined to accept the vaccine, emphasizing the importance of social and environmental factors in shaping health behaviors.

Conversely, lower socioeconomic status and household wealth were associated with reduced acceptance, as evidenced by the lowest wealth index group showing an OR of 0.10. This finding underscores the need for targeted interventions that address the economic barriers to vaccine access, particularly in low‐ and middle‐income countries. The WTP for the malaria vaccine was moderate, with a pooled prevalence of 58.3%. The variability in WTP reflects the economic disparities that affect vaccine access, highlighting the critical need for policies that subsidize vaccine costs and ensure equitable access.

These findings align with the WHO emphasis on the significant impact and broad implementation of the RTS,S, malaria vaccine, which has been recommended for preventing malaria in children under five, based on its proven effectiveness in reducing disease burden [46, 47]. Since its deployment in 2019 in Ghana, Kenya, and Malawi, the RTS,S, vaccine has led to a 13% reduction in child mortality and significant decreases in severe malaria cases [48]. Phase 3 trials further demonstrated that the vaccine reduced malaria cases by over 50% in the first year, maintaining a 40% reduction over 4 years [48, 49]. The vaccine has shown a good safety profile, high community demand, and feasibility for widespread use without negatively impacting other prevention methods like insecticide‐treated bed nets [16].

This meta‐analysis supports the findings of previous studies. A review by Sulaiman et al. [20] found high caregiver acceptance of malaria vaccines for children under five in low‐ and middle‐income countries (LMICs), with an overall acceptance rate of 95.3% (95% CI: 93.0%–97.2%). The highest acceptance rates were reported in Nigeria (97.6%), followed by Ghana (94.6%) and Tanzania (92.5%). Key sociodemographic factors influencing acceptance in their study, such as place of residence, tribe, and education level, align with the review findings that demographic and socioeconomic variables significantly impact vaccine acceptance. However, their study also identified concerns about vaccine safety, efficacy, and a general lack of awareness as barriers to broader acceptance, which parallel the hesitancy factors identified in this meta‐analysis.

Similarly, another review by Mumtaz et al. [50] highlighted critical challenges related to the acceptance, availability, and feasibility of the RTS,S, malaria vaccine in sub‐Saharan Africa, which resonate with the logistical and infrastructural barriers identified in this review. They emphasized limited production capacity and the significant challenges posed by logistical constraints in remote areas, findings that align with the meta‐analysis of barriers to vaccine acceptance. Additionally, their observations about concerns regarding vaccine side effects and lower efficacy in younger infants are consistent with the hesitancy factors indicated in this review. Further supporting this, Chutiyami et al. [51] reviewed the efficacy, safety, and community perceptions of malaria vaccines in Africa and found variability in vaccine effectiveness and acceptance rates. Their findings of mixed community perceptions and the need for increased community engagement and improved logistics underscore the importance of addressing these factors to ensure the successful widespread adoption of malaria vaccines, as also suggested by this review study.

### 5.2 Limitations of the Evidence Included in the Review

This systematic review and meta‐analysis were conducted with rigorous methodologies, but several limitations warrant careful interpretation of the results. It is important to note that some factors linked to malaria vaccine acceptance did not achieve statistical significance. The potential reasons for this include small sample sizes in certain studies, variability in measurement techniques, and the complex nature of behaviors and attitudes towards vaccine acceptance. Furthermore, the majority of the included studies were conducted in sub‐Saharan Africa, which may not accurately represent other malaria‐endemic regions. Therefore, caution is advised when generalizing these findings globally.

Significant heterogeneity was observed among the studies, indicated by high I² values for awareness, acceptance, hesitancy, and WTP. This variability likely stems from differences in study populations, methodologies, or regional factors, potentially affecting the consistency and generalizability of the results. Although a sensitivity analysis was performed for vaccine acceptance, similar analyses for vaccine hesitancy, WTP, and other influential factors were not feasible due to the limited number of studies addressing these outcomes. The presence of publication bias, suggested by funnel plot asymmetry and an LFK index of 5.3, also raises concerns about the potential overestimation of the pooled prevalence estimates, possibly due to the preferential publication of studies with positive results. The exclusion of non‐English language studies might have omitted relevant data from certain malaria‐endemic regions, introducing a language bias. Lastly, the reliance on self‐reported measures in many studies might lead to response bias, affected by social desirability and recall biases, which could impact the accuracy of the reported rates for vaccine acceptance, hesitancy, and WTP. These factors should be considered when evaluating the findings of this review.

### 5.3 Limitations of the Review Processes Used

The review processes also have limitations that must be acknowledged. While the review followed PRISMA guidelines and employed rigorous methodologies for data extraction and analysis, the significant heterogeneity observed across studies indicates that the pooled estimates should be interpreted with caution. Although random‐effects models were used to address heterogeneity, the underlying variability between studies remains a challenge that cannot be fully mitigated by statistical methods alone.

Moreover, sensitivity analyses confirmed the robustness of the findings, showing that no single study disproportionately influenced the overall results. However, the presence of high heterogeneity suggests that there may be unaccounted‐for factors influencing the results. This limitation underscores the importance of considering the context and specific conditions of each study when interpreting the pooled estimates. Future research should aim to standardize methodologies and reporting practices to reduce heterogeneity and improve the comparability of studies on malaria vaccine acceptance.

### 5.4 Implications of the Results for Practice, Policy, and Future Research

The findings of this study have significant implications for practice, policy, and future research. The acceptance rates observed indicate a general readiness among populations to embrace the malaria vaccine. However, the moderate levels of awareness and hesitancy highlight areas that require targeted interventions. Effective communication strategies tailored to address specific concerns and misconceptions are essential to improve awareness and reduce hesitancy. Public health campaigns should focus on providing clear, transparent information about the benefits and safety of the malaria vaccine, leveraging trusted community figures to build confidence and trust [52, 53]. While the RTS,S malaria vaccine is currently provided at no cost to individuals through government and donor support, addressing economic barriers remains crucial for ensuring equitable and sustained vaccine access. Long‐term policies should focus on securing funding from governments and organizations like Gavi to ensure that vaccines remain affordable and accessible to all segments of the population.

Future research should focus on conducting longitudinal studies to monitor changes in vaccine acceptance and hesitancy over time. These studies would offer a deeper understanding of the dynamics that influence vaccine acceptance in various contexts and help identify the most effective interventions for improving vaccination rates. Additionally, it is important to explore the long‐term impact of the RTS,S, malaria vaccine on public health outcomes and investigate the evolving factors that may affect vaccine acceptance as the vaccine becomes more widely available. Understanding these trends will be critical for adapting strategies to maintain high vaccination rates and addressing any emerging challenges.

The findings from this review offer essential insights into the determinants of malaria vaccine acceptance, which are critical for enhancing vaccination strategies in endemic regions. By addressing the identified barriers and leveraging the key determinants of vaccine acceptance, policymakers and health practitioners can design more effective approaches to combat malaria and improve public health outcomes globally. The successful implementation of these strategies has the potential to significantly reduce malaria incidence and mortality, ultimately contributing to the global goal of malaria eradication.

## 6 Conclusion

This systematic review and meta‐analysis revealed an acceptance rate of 83% for the malaria vaccine, with variability noted in awareness (32%), hesitancy (14%), and willingness to pay (58%). Key determinants of acceptance included age, employment, and socioeconomic status. However, these results must be interpreted with caution due to considerable limitations such as publication bias and high heterogeneity. The predominance of studies from sub‐Saharan Africa and the absence of non‐English studies may limit the generalizability of these findings to other malaria‐endemic areas. These findings underscore the need for targeted interventions to enhance vaccine acceptance in regions with lower acceptance. Further research is required to explore a broader range of factors influencing vaccine acceptance, including awareness, hesitancy, and financial willingness, to support effective malaria eradication strategies globally.

## Author Contributions

**Ganesh Bushi:** conceptualization, data curation, formal analysis, validation. **Mahalaqua Nazli Khatib:** data curation, supervision, validation, writing – original draft, writing – review and editing. **Renuka Jyothi S.:** investigation, resources, supervision, validation, writing – review and editing. **Irwanjot Kaur:** formal analysis, resources, software. **Abhishek Sharma:** conceptualization, data curation, resources. **Suhaib Iqbal:** investigation, resources; validation, writing – original draft, writing – review and editing. **M. Ravi Kumar:** methodology, project administration. **Ashish Singh Chauhan:** conceptualization, data curation, supervision, writing – review and editing. **Teena Vishwakarma:** conceptualization, data curation, supervision. **Praveen Malik:** resources, software. **Quazi Syed Zahiruddin:** conceptualization, data curation, visualization, writing – original draft. **Mahendra Pratap Singh:** resources, validation, writing – review & editing. **Muhammed Shabil:** conceptualization, resources, supervision. **Sanjit Sah:** conceptualization, data curation, validation. **Tarek Sulaiman:** resources, software, validation. **Nawal A. Al Kaabi:** conceptualization, resources, writing – original draft. **Hayam A. Alrasheed:** data curation, software, writing – review and editing. **Mubarak Alfaresi:** investigation, visualization. **Eman Alamri:** Investigation, writing – review and editing. **Maha F. Al‐Subaie:** Conceptualization, data curation, writing – review and editing. **Ali A. Rabaan:** conceptualization, resources.

## Ethics Statement

The authors have nothing to report.

## Conflicts of Interest

The authors declare no conflicts of interest.

## Supporting information

Figure S1. Forest plot depicting the factors influencing malaria vaccine acceptance.
Figure S2. Leave‐one‐out plot showing the results of sensitivity analysis.
Figure S3.
DOI plot illustrating publication bias.
Figure S4. Leave‐one‐out plot showing the results of sensitivity analysis of vaccine acceptance.2.

PRISMA checklist.

## Data Availability

All data generated or analyzed during this study are included in this published article (and its Supporting Information files).
