# Supplementary material for: Determinants of Malaria Vaccine Acceptance: A Systematic Review and Meta‐Analysis of Awareness, Acceptance, Hesitancy, and Willingness to Pay
Source: Immun Inflamm Dis. 2025 May 14;13(5):e70205. doi: 10.1002/iid3.70205 (PMC12076350; doi:10.1002/iid3.70205)
Supplement: Supplementary file 1 — Figure S1. Forest plot depicting the factors influencing malaria vaccine acceptance. Figure S2. Leave‐one‐out plot showing the results of sensitivity analysis. Figure S3. DOI plot illustrating publication bias. Figure S4. Leave‐one‐out plot showing the results of sensitivity analysis of vaccine acceptance.2. [file IID3-13-e70205-s001.docx]

**Supplementary Materials**


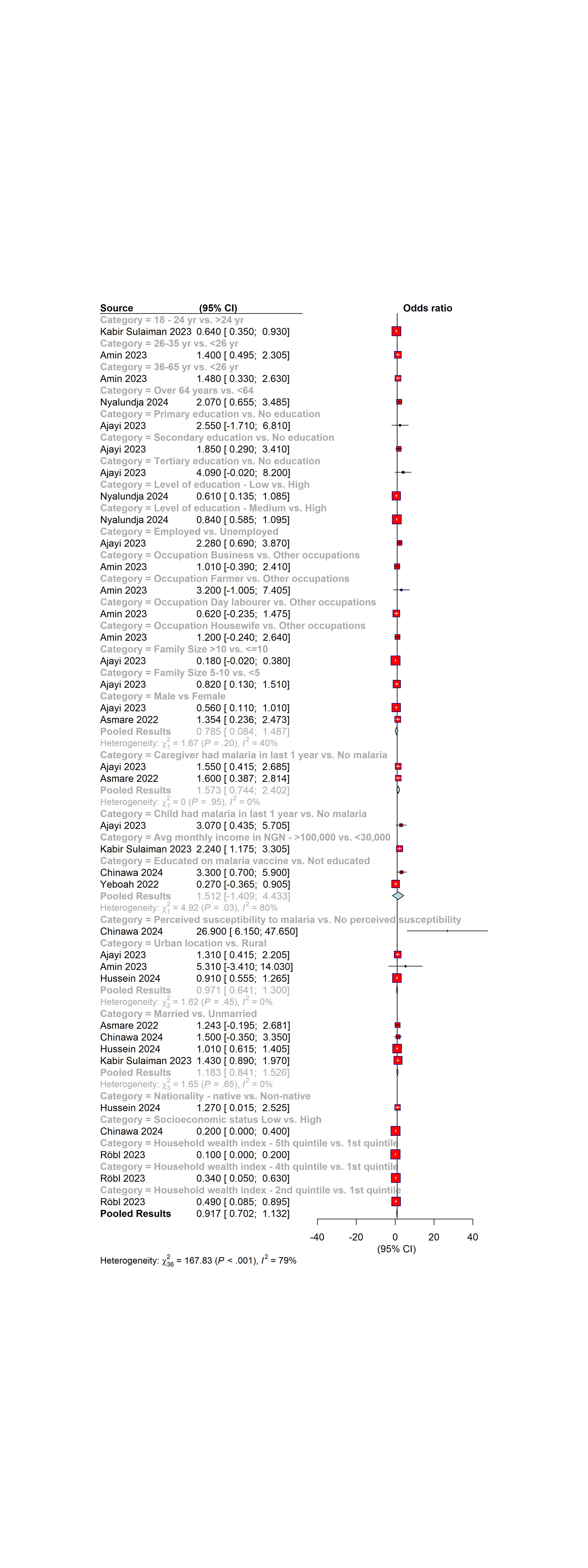


**Figure S1.** Forest plot depicting the factors influencing malaria vaccine acceptance.


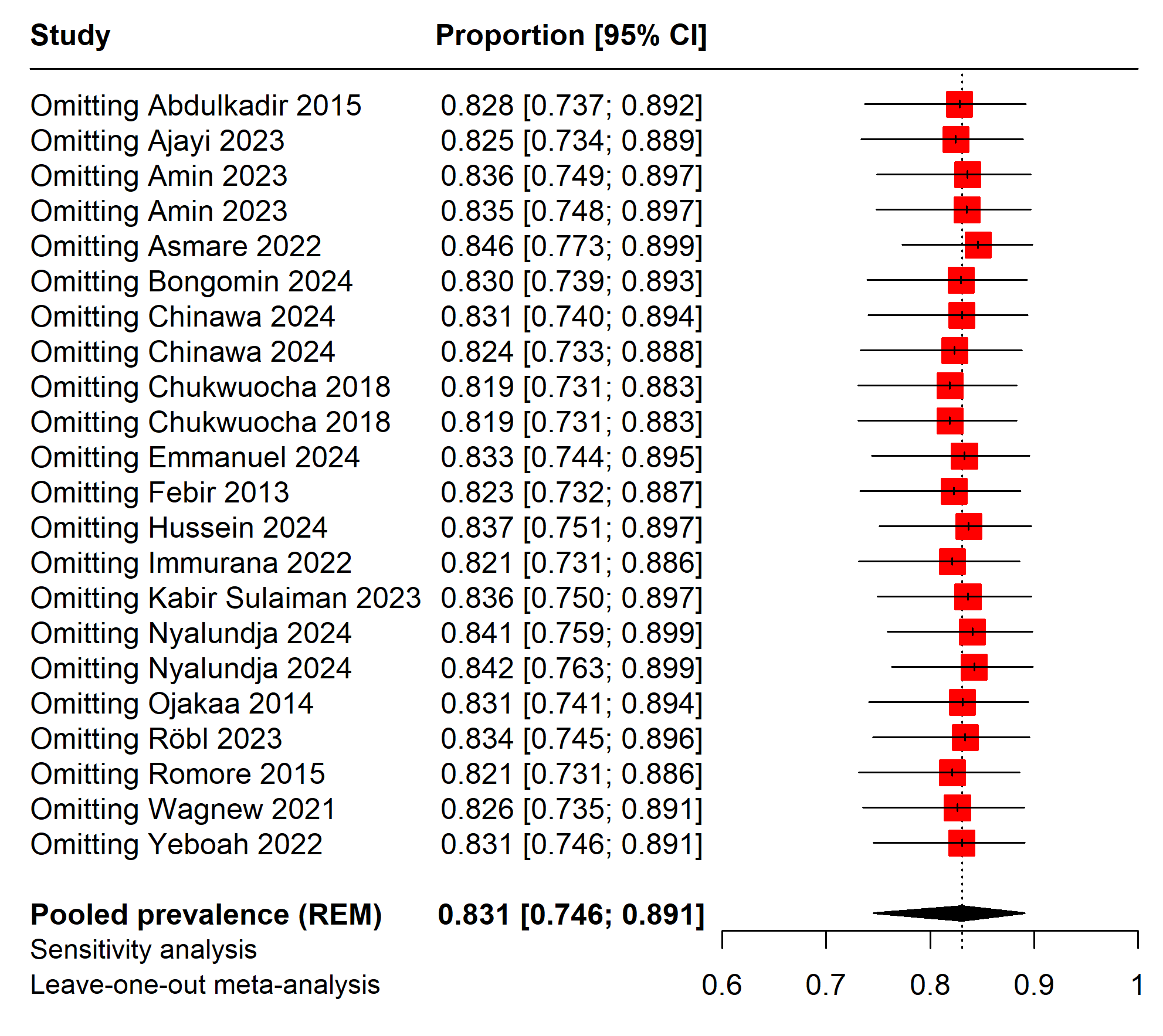


**Figure S2.** Leave-one-out plot showing the results of sensitivity analysis of vaccine acceptance.


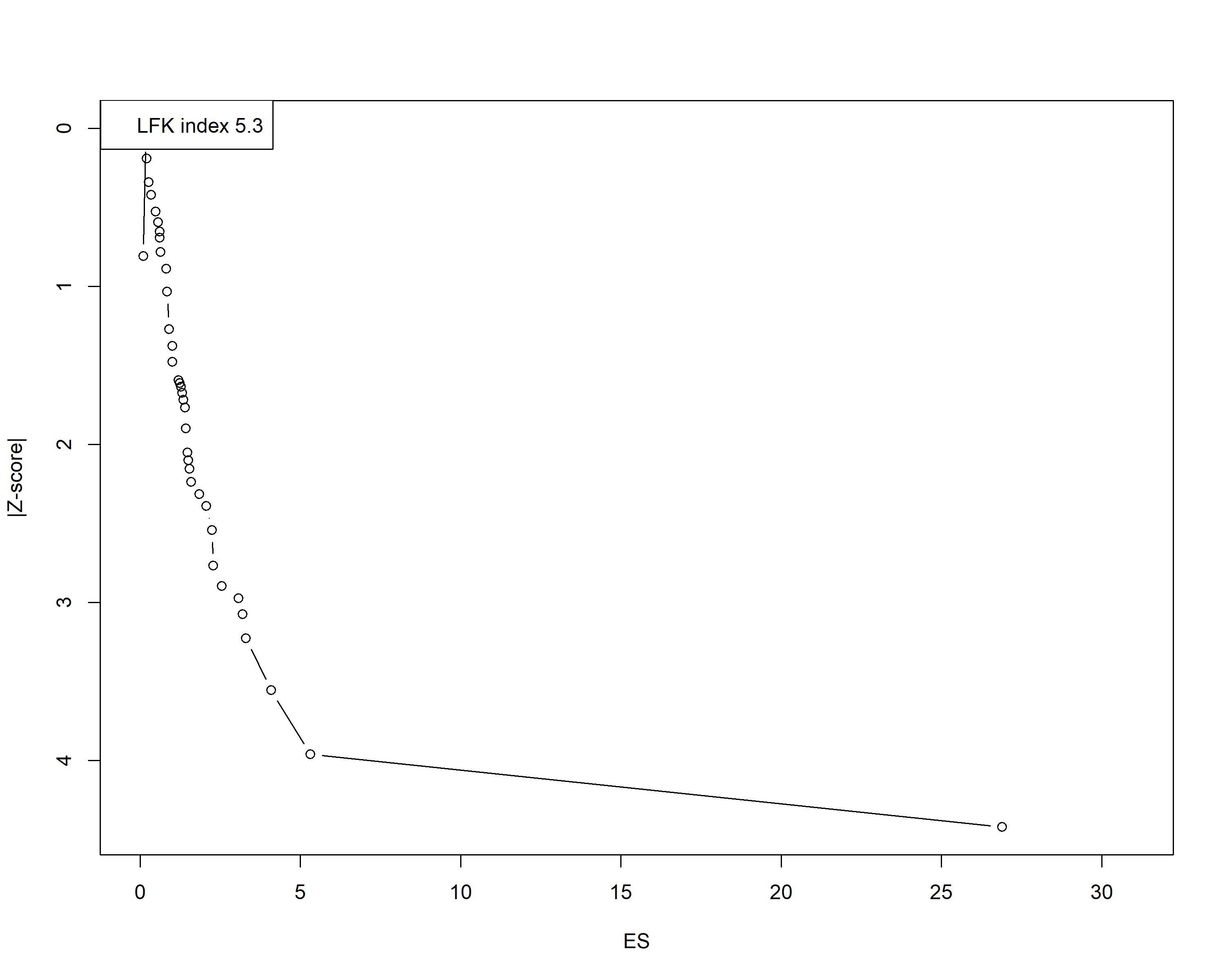


**Figure S3.** DOI plot illustrating publication bias

## **Table S1.** PRISMA Checklist

| **Section and Topic** | **Item #** | **Checklist item** | **Page no. where item is reported** |
| --- | --- | --- | --- |
| **TITLE** | | |  |
| Title | 1 | Identify the report as a systematic review. | 1 |
| **ABSTRACT** | | |  |
| Abstract | 2 | See the PRISMA 2020 for Abstracts checklist. (made as per the Journal guidelines) | 3 |
| **INTRODUCTION** | | |  |
| Rationale | 3 | Describe the rationale for the review in the context of existing knowledge. | 4 |
| Objectives | 4 | Provide an explicit statement of the objective(s) or question(s) the review addresses. | 5 |
| **METHODS** | | |  |
| Eligibility criteria | 5 | Specify the inclusion and exclusion criteria for the review and how studies were grouped for the syntheses. | 5, Table S2 |
| Information sources | 6 | Specify all databases, registers, websites, organisations, reference lists and other sources searched or consulted to identify studies. Specify the date when each source was last searched or consulted. | 5 |
| Search strategy | 7 | Present the full search strategies for all databases, registers and websites, including any filters and limits used. | 5, Table S3 |
| Selection process | 8 | Specify the methods used to decide whether a study met the inclusion criteria of the review, including how many reviewers screened each record and each report retrieved, whether they worked independently, and if applicable, details of automation tools used in the process. | 4 |
| Data collection process | 9 | Specify the methods used to collect data from reports, including how many reviewers collected data from each report, whether they worked independently, any processes for obtaining or confirming data from study investigators, and if applicable, details of automation tools used in the process. | 5,6 |
| Data items | 10a | List and define all outcomes for which data were sought. Specify whether all results that were compatible with each outcome domain in each study were sought (e.g., for all measures, time points, analyses), and if not, the methods used to decide which results to collect. | 6 |
|  | 10b | List and define all other variables for which data were sought (e.g., participant and intervention characteristics, funding sources). Describe any assumptions made about any missing or unclear information. | 6 |
| Study risk of bias assessment | 11 | Specify the methods used to assess risk of bias in the included studies, including details of the tool(s) used, how many reviewers assessed each study and whether they worked independently, and if applicable, details of automation tools used in the process. | 6, Table S4 |
| Effect measures | 12 | Specify for each outcome the effect measure(s) (e.g. risk ratio, mean difference) used in the synthesis or presentation of results. | 6 |
| Synthesis methods | 13a | Describe the processes used to decide which studies were eligible for each synthesis (e.g. tabulating the study intervention characteristics and comparing against the planned groups for each synthesis (item #5)). | 6 |
|  | 13b | Describe any methods required to prepare the data for presentation or synthesis, such as handling of missing summary statistics, or data conversions. | NA |
|  | 13c | Describe any methods used to tabulate or visually display results of individual studies and syntheses. |  |
|  | 13d | Describe any methods used to synthesize results and provide a rationale for the choice(s). If meta-analysis was performed, describe the model(s), method(s) to identify the presence and extent of statistical heterogeneity, and software package(s) used. | 6 |
|  | 13e | Describe any methods used to explore possible causes of heterogeneity among study results (e.g. subgroup analysis, meta-regression). | 6 |
|  | 13f | Describe any sensitivity analyses conducted to assess robustness of the synthesized results. | 6 |
| Reporting bias assessment | 14 | Describe any methods used to assess risk of bias due to missing results in a synthesis (arising from reporting biases). | 6 |
| Certainty assessment | 15 | Describe any methods used to assess certainty (or confidence) in the body of evidence for an outcome. | NA |
| **RESULTS** | | |  |
| Study selection | 16a | Describe the results of the search and selection process, from the number of records identified in the search to the number of studies included in the review, ideally using a flow diagram. | 7 |
|  | 16b | Cite studies that might appear to meet the inclusion criteria, but which were excluded, and explain why they were excluded. | NA |
| Study characteristics | 17 | Cite each included study and present its characteristics. | 7 Table 1 |
| Risk of bias in studies | 18 | Present assessments of risk of bias for each included study. | Table S4 |
| Results of individual studies | 19 | For all outcomes, present, for each study: (a) summary statistics for each group (where appropriate) and (b) an effect estimate and its precision (e.g. confidence/credible interval), ideally using structured tables or plots. | Table 1, Figure 2,3,4 |
| Results of syntheses | 20a | For each synthesis, briefly summarise the characteristics and risk of bias among contributing studies. | 7, 8 |
|  | 20b | Present results of all statistical syntheses conducted. If meta-analysis was done, present for each the summary estimate and its precision (e.g. confidence/credible interval) and measures of statistical heterogeneity. If comparing groups, describe the direction of the effect. | 5,4 Figure 2 |
|  | 20c | Present results of all investigations of possible causes of heterogeneity among study results. | Figure S2 |
|  | 20d | Present results of all sensitivity analyses conducted to assess the robustness of the synthesized results. | Figure S1 |
| Reporting biases | 21 | Present assessments of risk of bias due to missing results (arising from reporting biases) for each synthesis assessed. | NA |
| Certainty of evidence | 22 | Present assessments of certainty (or confidence) in the body of evidence for each outcome assessed. | NA |
| **DISCUSSION** | | |  |
| Discussion | 23a | Provide a general interpretation of the results in the context of other evidence. | 9, 10 |
|  | 23b | Discuss any limitations of the evidence included in the review. | 11 |
|  | 23c | Discuss any limitations of the review processes used. | 12 |
|  | 23d | Discuss implications of the results for practice, policy, and future research. | 12, 13 |
| **OTHER INFORMATION** | | |  |
| Registration and protocol | 24a | Provide registration information for the review, including register name and registration number, or state that the review was not registered. | 5 |
|  | 24b | Indicate where the review protocol can be accessed, or state that a protocol was not prepared. | 5 |
|  | 24c | Describe and explain any amendments to information provided at registration or in the protocol. | NA |
| Support | 25 | Describe sources of financial or non-financial support for the review, and the role of the funders or sponsors in the review. | 14 |
| Competing interests | 26 | Declare any competing interests of review authors. | 14 |
| Availability of data, code and other materials | 27 | Report which of the following are publicly available and where they can be found: template data collection forms; data extracted from included studies; data used for all analyses; analytic code; any other materials used in the review. | Supplementary Materials |

**Table S2.** Inclusion and Exclusion criteria

|  | Inclusion | Exclusion |
| --- | --- | --- |
| Participants | Individuals from malaria-endemic regions | NA |
| Exposure | Malaria vaccine | Not information of the malaria vaccine |
| Outcome | Awareness, acceptance, hesitancy, willingness to pay (WTP) for the malaria vaccine | Safety, efficacy and immunogenicity |
| Study Designs | Observational studies, cross-sectional studies, cohort studies, case-control studies | Clinical trials, narrative reviews, commentaries, etc., |
| Geography | Global, focusing on malaria-endemic regions | NA |
| Date of Search | Publications up to 18 June 2024 | NA |
| Publication Type | Published articles and articles available in English | Unpublished and articles not available in English |

**Table S3. The adjusted search terms as per searched electronic databases.**

| Database | No | Search Query | Results |
| --- | --- | --- | --- |
| PubMed | #1 | (((((((Malaria[Title/Abstract]) OR (Remittent Fever[Title/Abstract])) OR (Paludism[Title/Abstract])) OR (marsh fever[Title/Abstract])) OR (Plasmodium[Title/Abstract])) OR (plasmodium infection[Title/Abstract])) OR ("malaria"[MeSH Terms] OR "malaria"[Title/Abstract] OR "malarias"[Title/Abstract] OR "malaria s"[Title/Abstract] OR "malariae"[Title/Abstract])) OR ("plasmodium falciparum"[MeSH Terms] OR ("plasmodium"[Title/Abstract] AND "falciparum"[Title/Abstract]) OR "plasmodium falciparum"[Title/Abstract]) OR ("plasmodium vivax"[MeSH Terms] OR ("plasmodium"[Title/Abstract] AND "vivax"[Title/Abstract]) OR "plasmodium vivax"[Title/Abstract]) | 121,722 |
|  | #2 | **((((((((((vaccine acceptance[Title/Abstract]) OR (vaccine hesitancy[Title/Abstract])) OR (vaccine hesitancy[Title/Abstract])) ) OR (vaccine delay[Title/Abstract])) OR (vaccine refusal[Title/Abstract])) OR (Vaccination hesitancy[Title/Abstract])) OR (Vaccination acceptance[Title/Abstract])) OR (vaccine accept[Title/Abstract])) OR (vaccine hesit*[Title/Abstract])) OR ("Acceptance"[Title/Abstract] OR "Willingness"[Title/Abstract] OR "Unwillingness"[Title/Abstract] OR "Hesitancy"[Title/Abstract] OR "Intention"[Title/Abstract] OR "Unacceptance"[Title/Abstract])** | 215,810 |
|  | #5 | #1 AND #2 | 825 |
| EMBASE | #1 | Malaria:ab,ti OR ‘Remittent Fever’:ab,ti OR ‘Paludism’:ab,ti OR ‘marsh fever’:ab,ti OR Plasmodium:ab,ti OR ‘plasmodium infection’:ab,ti OR malaria:ab,ti OR malarias:ab,ti OR malariae:ab,ti OR ‘plasmodium falciparum’:ab,ti OR plasmodium:ab,ti AND falciparum:ab,ti OR ‘plasmodium falciparum’:ab,ti OR ‘plasmodium vivax’:ab,ti OR plasmodium:ab,ti AND vivax:ab,ti OR ‘plasmodium vivax’:ab,ti | 11458 |
|  | #2 | ‘vaccine acceptance’:ab,ti OR ‘vaccine hesitancy’:ab,ti OR ‘vaccine hesitancy’:ab,ti OR ‘vaccine delay’:ab,ti OR ‘vaccine refusal’:ab,ti OR ‘Vaccination hesitancy’:ab,ti OR ‘Vaccination acceptance’:ab,ti OR ‘vaccine accept’:ab,ti OR ‘vaccine hesit’:ab,ti OR ‘Acceptance’:ab,ti OR ‘Willingness’:ab,ti OR ‘Unwillingness’:ab,ti OR ‘Hesitancy’:ab,ti OR ‘Intention’:ab,ti OR ‘Unacceptance’:ab,ti | 280746 |
|  | #5 | #1 AND #2 | 91 |
| WOS  advanced | #1 | (TI=(Malaria OR Remittent Fever OR Paludism OR marsh fever OR Plasmodium OR plasmodium infection OR malaria OR malaria OR malarias OR malaria s OR malariae OR plasmodium falciparum OR plasmodium AND falciparum OR plasmodium falciparum OR plasmodium vivax OR plasmodium AND vivax OR plasmodium vivax)) OR AB=(Malaria OR Remittent Fever OR Paludism OR marsh fever OR Plasmodium OR plasmodium infection OR malaria OR malaria OR malarias OR malaria s OR malariae OR plasmodium falciparum OR plasmodium AND falciparum OR plasmodium falciparum OR plasmodium vivax OR plasmodium AND vivax OR plasmodium vivax) | 110404 |
|  | #2 | TI=(vaccine acceptance OR vaccine hesitancy OR vaccine hesitancy OR vaccine delay OR vaccine refusal OR Vaccination hesitancy OR Vaccination acceptance OR vaccine accept OR vaccine hesit* OR Acceptance OR Willingness OR Unwillingness OR Hesitancy OR Intention OR Unacceptance)) OR AB=(vaccine acceptance OR vaccine hesitancy OR vaccine hesitancy OR vaccine delay OR vaccine refusal OR Vaccination hesitancy OR Vaccination acceptance OR vaccine accept OR vaccine hesit* OR Acceptance OR Willingness OR Unwillingness OR Hesitancy OR Intention OR Unacceptance) | 250190 |
|  | #5 | #1 AND #2 | 900 |

**Table S4.** Modified Newcastle-Ottawa Scale tool for the quality assessment of studies

| **Sl.no** | **Study** | **Representativeness of population** | **Sample size 500** | **Definition** | **Ascertainment of outcomes** | **Total** |
| --- | --- | --- | --- | --- | --- | --- |
|  | Abdulkadir 2015 (1) | 1 | 1 | 1 | 1 | 4 |
|  | Ajayi 2023 (2) | 2 | 1 | 2 | 1 | 6 |
|  | Amin 2023 (3) | 2 | 1 | 1 | 1 | 5 |
|  | Asmare 2022 (4) | 1 | 1 | 2 | 1 | 5 |
|  | Bongomin 2024 (5) | 1 | 1 | 1 | 1 | 4 |
|  | Chinawa 2024 (6) | 2 | 0 | 2 | 1 | 5 |
|  | Chukwuocha 2018 (7) | 1 | 1 | 1 | 1 | 4 |
|  | Emmanuel 2024 (8) | 2 | 1 | 1 | 1 | 5 |
|  | Febir 2013 (9) | 2 | 1 | 2 | 1 | 6 |
|  | Hussein 2024 (10) | 2 | 1 | 1 | 1 | 5 |
|  | Immurana 2022 (11) | 1 | 1 | 1 | 1 | 4 |
|  | Kabir Sulaiman 2023 (12) | 2 | 0 | 2 | 1 | 5 |
|  | Nyalundja 2024 (13) | 1 | 1 | 1 | 1 | 4 |
|  | Ojakaa 2014 (14) | 1 | 1 | 1 | 1 | 4 |
|  | Röbl 2023 (15) | 2 | 0 | 2 | 1 | 5 |
|  | Romore 2015 (16) | 1 | 1 | 1 | 1 | 4 |
|  | Wagnew 2021 (17) | 2 | 1 | 1 | 1 | 5 |
|  | Yeboah 2022 (18) | 2 | 1 | 2 | 1 | 6 |

**Bibliography**

1. Abdulkadir BI, Ajayi IO. Willingness to accept malaria vaccine among caregivers of under-5 children in Ibadan North Local Government Area, Nigeria. MalariaWorld J. 2015;6(2).

2. Ajayi MY, Emeto DC. Awareness and acceptability of malaria vaccine among caregivers of under-5 children in Northern Nigeria. Malaria Journal. 2023;22(1):329.

3. Amin MA, Afrin S, Bonna AS, Rozars MFK, Nabi MH, Hawlader MDH. Knowledge and acceptance of malaria vaccine among parents of under‐five children of malaria endemic areas in Bangladesh: A cross‐sectional study. Health Expectations. 2023;26(6):2630-43.

4. Asmare G. Willingness to accept malaria vaccine among caregivers of under-5 children in Southwest Ethiopia: a community based cross-sectional study. Malaria Journal. 2022;21(1):146.

5. Bongomin F, Megwera FJ, Mundua J, Naluwooza N, Ayesiga F, Nsubuga Y, et al. Malaria vaccine acceptance among next of kin of children under 5 years of age in Gulu, northern Uganda in 2023: a community-based study. Therapeutic Advances in Infectious Disease. 2024;11:20499361241247467.

6. Chinawa AT, Ossai EN, Onukwuli VO, Nduagubam OC, Uwaezuoke NA, Okafor CN, Chinawa JM. Willingness to accept malaria vaccines amongst women presenting at outpatient and immunization clinics in Enugu state, Southeast Nigeria. Malaria Journal. 2024;23(1):117.

7. Chukwuocha UM, Okorie PC, Iwuoha GN, Ibe SN, Dozie IN, Nwoke BE. Awareness, perceptions and intent to comply with the prospective malaria vaccine in parts of South Eastern Nigeria. Malaria Journal. 2018;17:1-7.

8. Emmanuel BN, Ishaq AN, Akunne OZ, Saidu UF. Evaluating the knowledge, attitude, perception, and readiness of caregivers of under 5-year-old children to accept malaria vaccine in Nigeria. Clinical and Experimental Vaccine Research. 2024;13(2):121.

9. Febir LG, Asante KP, Dzorgbo D-BS, Senah KA, Letsa TS, Owusu-Agyei S. Community perceptions of a malaria vaccine in the Kintampo districts of Ghana. Malaria Journal. 2013;12:1-10.

10. Hussein MF, Kyei-Arthur F, Saleeb M, Kyei-Gyamfi S, Abutima T, Sakada IG, Ghazy RM. Hesitancy towards R21/Matrix-M malaria vaccine among Ghanaian parents and attitudes towards immunizing non-eligible children: a cross-sectional survey. Malaria Journal. 2024;23(1):142.

11. Immurana M, Boachie MK, Klu D, Dalaba MA, Manyeh AK, Alhassan RK. Determinants of willingness to accept child vaccination against malaria in Ghana. The International Journal of Health Planning and Management. 2022;37(3):1439-53.

12. Sulaiman SK, Tsiga-Ahmed FIi, Musa MS, Sulaiman AK, Dayyab FM, Khan MA, et al. Prevalence, determinants, and reasons for malaria vaccine hesitancy among caregivers of under-five children in Nigeria: results from a nationwide cross-sectional survey. Vaccine. 2023;41(8):1503-12.

13. Nyalundja AD, Bugeme PM, Guillaume AS, Ntaboba AB, Hatu’m VU, Tamuzi JL, et al. Socio-Demographic Factors Influencing Malaria Vaccine Acceptance for Under-Five Children in a Malaria-Endemic Region: A Community-Based Study in the Democratic Republic of Congo. Vaccines. 2024;12(4):380.

14. Ojakaa DI, Jarvis JD, Matilu MI, Thiam S. Acceptance of a malaria vaccine by caregivers of sick children in Kenya. Malaria Journal. 2014;13:1-12.

15. Röbl K, Fischer H-T, Delamou A, Mbawah AK, Geurts B, Feddern L, et al. Caregiver acceptance of malaria vaccination for children under 5 years of age and associated factors: cross-sectional household survey, Guinea and Sierra Leone, 2022. Malaria Journal. 2023;22(1):355.

16. Romore I, Ali AM, Semali I, Mshinda H, Tanner M, Abdulla S. Assessment of parental perception of malaria vaccine in Tanzania. Malaria journal. 2015;14:1-6.

17. Wagnew Y, Hagos T, Weldegerima B, Debie A. Willingness to pay for childhood malaria vaccine among caregivers of under-five children in Northwest Ethiopia. ClinicoEconomics and Outcomes Research. 2021:165-74.

18. Yeboah D, Owusu-Marfo J, Agyeman YN. Predictors of malaria vaccine uptake among children 6–24 months in the Kassena Nankana Municipality in the Upper East Region of Ghana. Malaria Journal. 2022;21(1):339.
